# The AGE–RAGE–DIAPH1 Axis in Type 2 Diabetes and Metabolic Dysfunction: From Carbonyl Stress to Diabetic Myocardial and Neuronal Injury

**DOI:** 10.3390/ijms27125305

**Published:** 2026-06-11

**Authors:** Bernard Kordas, Judyta Juranek

**Affiliations:** Department of Human Physiology and Pathophysiology, School of Medicine, Collegium Medicum, University of Warmia and Mazury, 10-082 Olsztyn, Poland

**Keywords:** advanced glycation end-products, RAGE, DIAPH1, carbonyl stress, type 2 diabetes mellitus, diabetic myocardial disorder, cardiovascular autonomic neuropathy, neurodegeneration

## Abstract

Carbonyl stress, chronic inflammation, and progressive tissue injury accompany type 2 diabetes mellitus (T2DM) and obesity. Yet, the molecular systems that connect these processes with cardiac, vascular and neuronal complications are incompletely defined. This review examines the AGE–RAGE–DIAPH1 axis as a mechanistic link between metabolic dysfunction and diabetic myocardial and neuronal injury, with emphasis on vascular and myocardial remodeling and emerging implications for autonomic neuronal vulnerability. We summarize current evidence on the formation and accumulation of advanced glycation end-products and other RAGE ligands in metabolic disease, DIAPH1’s structural and signaling role as an intracellular effector of RAGE, and the cellular consequences of pathway activation in vascular, neural, and cardiac tissues. Across experimental models, this signaling axis promotes oxidative stress and inflammatory activation, leading to endothelial dysfunction and barrier failure. Subsequent fibrotic remodeling provides a biologically plausible route through which metabolic stress may be translated into persistent organ injury. In the heart, these mechanisms are linked to coronary microvascular dysfunction, altered cardiomyocyte phenotype, calcium handling abnormalities, and myocardial fibrosis. In the autonomic nervous system, limited but emerging data connect RAGE activation to oxidative injury and mitochondrial dysfunction, abnormal neuronal excitability, and structural vulnerability. Direct evidence linking DIAPH1 to autonomic neurons is lacking. We also review biomarker candidates related to this pathway, including circulating AGEs and soluble RAGE isoforms, skin AGE measurements, imaging markers of myocardial remodeling, and autonomic functional measures. Finally, we discuss pharmacological and natural compounds that target AGE formation, ligand accumulation, RAGE signaling, or intracellular protein interactions linked to this axis. Overall, the available evidence supports the AGE–RAGE–DIAPH1 axis as a credible mechanistic concept and a potentially informative translational hypothesis in T2DM. However, the AGE–RAGE component is supported more strongly than DIAPH1-specific involvement in human diabetic myocardial disorder or cardiovascular autonomic neuropathy. The value of DIAPH1 as a biomarker or therapeutic target in these neurocardiac complications remains to be established.

## 1. Introduction

In obesity and type 2 diabetes mellitus (T2DM), metabolic dysfunction emerges in the setting of chronic systemic inflammation [1,2]. Obesity is characterized by infiltration of adipose tissue by immune cells, particularly macrophages [3], as part of inflammatory remodeling associated with impaired insulin sensitivity and progressive metabolic dysfunction [4]. Given this, T2DM is more than a disorder of glycemic control, but also is a chronic condition in which insulin resistance and inflammation progressively damage vulnerable tissues [5].

Because it allows multiple metabolic and inflammatory signals to converge, the receptor for advanced glycation end-products (RAGE) assumes a central role in this context. Although initially identified as a receptor for advanced glycation end-products (AGEs) [6], it also binds high-mobility group box 1 (HMGB1) and several members of the S100 and calgranulin families [7]. Activation of RAGE promotes oxidative stress and inflammatory responses, which contribute to vascular dysfunction and tissue remodeling [8]. This links metabolic stress to sustained pathogenic activity across organs [9]. Signal propagation relies on RAGE’s short cytoplasmic domain interacting with diaphanous-related formin 1 (DIAPH1), not on intrinsic kinase activity [10]. Through this interaction, events initiated at the cell surface are coupled to intracellular changes in cellular architecture and downstream pathways [11]. This mechanism has gained translational relevance with the development of small-molecule antagonists that interrupt RAGE–DIAPH1 signaling and reduce diabetic complications in experimental models [12]. These processes are particularly detrimental in tissues that are highly sensitive to chronic metabolic stress, including the heart and the autonomic nervous system. Contemporary clinical consensus recognizes diabetic myocardial disorder as a phenotype that cannot be explained fully by epicardial coronary artery disease, valvular disease, or pressure overload alone [13]. This review uses “diabetic myocardial disorder” for diabetes-related heart abnormalities not explained by coronary artery disease, valvular disease, or pressure overload; “diabetic cardiomyopathy” is used as a search term or when cited. Findings from mechanistic studies help explain how AGE–RAGE signaling is involved in myocardial stress and coronary microvascular dysfunction [13,14]. Cardiovascular autonomic neuropathy (CAN) is a clinically significant complication of diabetes [15]. Clinically, CAN is defined as impairment of cardiovascular autonomic control in diabetes, after other causes have been ruled out. According to the Toronto Consensus Panel, one abnormal cardiovagal test may indicate possible or early CAN, at least two abnormal cardiovagal tests support definite CAN, and orthostatic hypotension in addition to abnormal cardiovagal testing indicates severe CAN. Standard diagnostic assessment relies mainly on cardiovascular autonomic reflex tests, including heart-rate responses to deep breathing, standing, and the Valsalva maneuver; blood-pressure response to standing; and heart-rate variability as an additional measure [16,17,18]. It is associated with cardiovascular events, arrhythmic risk, and increased mortality [18,19]. Recent mechanistic work has linked autonomic neuronal injury in diabetes to the RAGE axis via mitochondrial dysfunction and intracellular stress pathways [20].

### 1.1. Scope of the Review and Hierarchy of Evidence

This narrative review evaluates whether DIAPH1, a RAGE cytoplasmic-domain effector, plausibly extends AGE–RAGE biology to diabetic myocardial and neuronal injury. Evidence levels vary across the pathway. Extensive experimental and clinical research supports AGE formation, RAGE ligand buildup, and RAGE-dependent oxidative and inflammatory signaling. By contrast, direct evidence implicating DIAPH1 in human diabetic myocardial disorder or cardiovascular autonomic neuropathy is limited. Accordingly, throughout this review, we distinguish between direct DIAPH1-specific evidence, evidence centered on RAGE without direct DIAPH1 validation, and AGE or carbonyl stress evidence. This distinction is essential because DIAPH1 should be regarded as a potential therapeutic target, not a confirmed clinical marker. The authors’ qualitative ratings reflect their interpretation of evidence strength and are not a formal grading system; they are not intended to function as a validated evidence-grading framework. Table 1 summarizes the relative strength of evidence across key domains considered in this review.

### 1.2. Literature Search Strategy

This article was written as a narrative mechanistic review. We searched PubMed, Scopus, Web of Science, and Google Scholar complemented by targeted Google searches for relevant guidelines, consensus documents, and source materials, up to 17 April 2026. Search terms included combinations of “advanced glycation end-products”, “AGEs”, “RAGE”, “DIAPH1”, “mDia1”, “type 2 diabetes”, “metabolic dysfunction”, “diabetic myocardial disorder”, “diabetic cardiomyopathy”, “cardiovascular autonomic neuropathy”, “heart rate variability”, “autonomic neurons”, “diabetic neuropathy”, “neuronal injury”, “neurodegeneration”, “microvascular dysfunction”, “fibrosis”, “RAGE antagonist”, “RAGE–DIAPH1 antagonist”, “natural compounds”, and “nutraceuticals”. Priority was given to recent studies, particularly those published from 2021 onward, when they provided relevant mechanistic, translational, or clinical evidence. Foundational studies were included when necessary to explain AGE formation, RAGE biology, DIAPH1 structure, or canonical signaling mechanisms.

**Figure 1:**
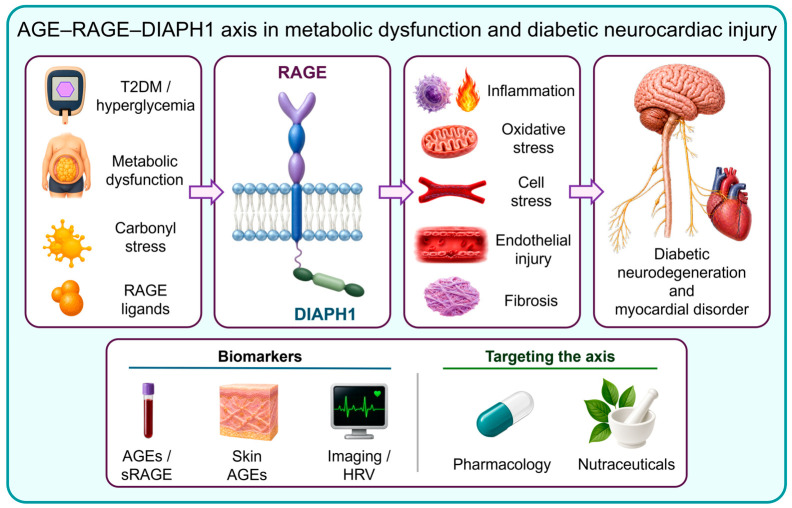
Schematic overview of the AGE–RAGE–DIAPH1 axis in diabetic neurocardiac injury. Impaired glucose control, metabolic dysfunction, and carbonyl stress increase the levels of RAGE ligands. RAGE activates DIAPH1-dependent signaling, causing inflammation and oxidative damage. This damages the endothelium and promotes fibrosis. Tissue and organ injury includes diabetic neuronal injury, autonomic involvement, and diabetic myocardial disorder. Biomarker readouts include circulating AGE/RAGE markers, skin AGE measurements, cardiac imaging, and heart rate variability. In contrast, therapeutic targeting encompasses pharmacological approaches and natural compounds that act at different levels of the axis. The figure is conceptual. Evidence for AGE–RAGE signaling is stronger than evidence for DIAPH1 involvement in human diabetic myocardial or neuronal disease.

Figure 1 provides an overview of the proposed AGE–RAGE–DIAPH1 axis in metabolic dysfunction, linking T2DM, carbonyl stress, and RAGE ligand accumulation with downstream cellular injury, biomarker readouts, therapeutic targeting, and damage to the microvasculature, myocardium, and autonomic nervous system.

## 2. Formation of AGE Ligands and RAGE–DIAPH1 Signaling in Metabolic Dysfunction

### 2.1. Dicarbonyl Generation and AGE Formation

When reactive carbonyl species are produced in excess and cannot be detoxified, they modify cellular macromolecules and cause metabolic injury [21]. In diabetes, methylglyoxal (MGO), glyoxal, and 3-deoxyglucosone are major dicarbonyl intermediates because they react with biological macromolecules faster than glucose does [22]. MGO forms through the spontaneous breakdown of glyceraldehyde 3-phosphate and dihydroxyacetone phosphate during glycolysis [23]. Sustained hyperglycemia increases its formation by expanding intracellular triose phosphate pools and associated glycolytic activity [24]. Insulin resistance and excess lipids increase dicarbonyl stress by inducing substrate overload and the oxidative breakdown of carbohydrates and lipids [22]. Plasma MGO is elevated in newly diagnosed T2DM [25] and, in established disease, predicts cardiovascular events and mortality [26].

### 2.2. Ligand Accumulation and RAGE Activation in Metabolic Dysfunction

**Figure 2:**
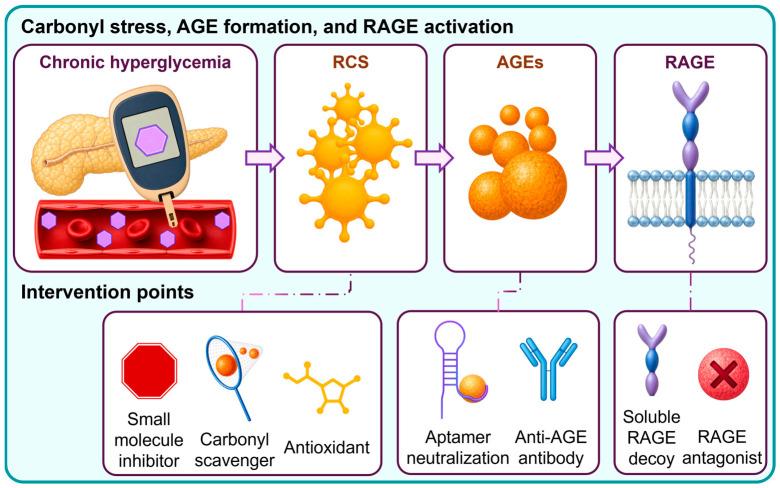
From carbonyl stress to receptor engagement—early activation of the AGE–RAGE axis and extracellular intervention points. Chronic hyperglycemia and metabolic dysfunction promote the generation of reactive carbonyl species (RCS), which accelerate the formation of advanced glycation end-products (AGEs). AGE ligands engage RAGE at the cell membrane and initiate receptor signaling. The lower part of the figure summarizes intervention points that act before or at the receptor, including small-molecule inhibitors of AGE formation, carbonyl scavengers, antioxidant approaches, aptamer-mediated neutralization of ligands, antibodies against AGEs, soluble RAGE decoy strategies, and RAGE antagonists.

AGEs form from early glycation products and mature into stable adducts and crosslinks. MGO-derived hydroimidazolone 1, Nε-carboxymethyllysine (CML), and glucosepane are commonly detected in vivo [27]. Because reactive dicarbonyls are more chemically active than glucose, they disproportionately drive AGE formation [22]. Extracellular matrix proteins with low turnover rates gather these alterations as they age. Human skin collagen in diabetes shows increased nonenzymatic glucosylation and decreased solubility [28]. Although strict glycemic control reduces glycoxidation and crosslinking, it does not fully prevent them [29]. AGE-modified extracellular matrix in peripheral nerves hinders neurite extension in vitro [30]. Figure 2 summarizes the pathway from chronic hyperglycemia to RAGE activation and highlights pharmacological intervention points.

In metabolic dysfunction, ligands presented to RAGE are not only AGEs. HMGB1 is increased in T2DM, and high glucose induces HMGB1 in mesangial cells [31]. HMGB1 also contributes to adipose tissue inflammation in obesity [32]. Members of the S100 family increase in metabolic disease. Circulating calprotectin is elevated in obesity regardless of T2DM status [33]. Blood cell expression of S100A8, S100A9, and S100A12 correlates with visceral adiposity, insulin resistance, and inflammation [34]. In metabolic dysfunction caused by obesity, RAGE expression increases in adipose tissue together with inflammatory and insulin resistance characteristics [35].

Proteins that contain CML engage RAGE and activate inflammatory signaling pathways that include NF-κB and extracellular signal-regulated kinase signaling [36]. In endothelial cells, AGE–RAGE leads to higher expression of adhesion molecules and triggers oxidative stress, together with inflammatory activation [37]. In diabetes, mononuclear phagocytes show sustained NF-κB activation [38]. RAGE-dependent signaling also increases superoxide generation in these cells [39].

In sensory neurons, RAGE activation triggers oxidative stress [40]. Studies suggest that RAGE contributes to diabetic damage in both neurons and heart cells. In sensory neurons, high glucose potentiates TRPV1 currents through RAGE [41], while diabetes is accompanied by increased RAGE expression across neural cell populations [42]. Hyperglycemia also affects autonomic neurons, producing RAGE-linked mitochondrial abnormalities [20]. Related changes are also seen in the diabetic heart. Endothelial and immune cells in diabetic hearts upregulate RAGE [43], and AGEs alter cardiomyocyte phenotype [44]. RAGE signaling drives cardiac fibroblasts’ migration and inflammatory responses [45].

### 2.3. DIAPH1 as an Intracellular Effector of RAGE Signaling Relevant to Neurocardiac Injury

RAGE signaling requires an intracellular effector to link its short cytoplasmic tail to downstream responses [11,46]. DIAPH1 (mDia1) is a diaphanous-related formin, originally identified as a Rho effector that binds profilin [47]. Domain mapping studies showed that DIAPH1 contains a Rho GTPase binding region, a proline-rich formin homology 1 region that binds profilins, and a C-terminal autoinhibitory module that constrains activity in the resting state [48]. mDia1 is inactive until activation signals remove its autoinhibition [49,50]. Structural analysis confirmed that active Rho binding frees the regulatory region and enables actin assembly [51]. These studies establish DIAPH1 as a regulated actin-organizing protein capable of converting receptor activation into changes in cellular architecture and behavior [49,51].

Early work showed that diaphanous-related formins serve as a connection between Rho GTPase and Src-family signaling. That places DIAPH1 between cytoskeletal remodeling and signal transduction [52]. DIAPH1 controls serum response factor activity by modulating actin polymerization. This provides a mechanism through which receptor engagement at the membrane can alter gene expression after ligand binding [53]. mDia1 is sufficient to induce the formation and orientation of stable microtubules in response to Rho [54]. This effect is coordinated through interactions with EB1 and APC, which stabilize microtubules and promote directed cell migration [55]. During cell migration, the pathway from Rho to mDia1 regulates cell polarity and focal adhesion turnover by aligning actin filaments with microtubules and mobilizing proteins required for polarized movement [56]. These studies show that DIAPH1 coordinates the two cytoskeletal systems that determine adhesion, polarity, and locomotion [54,55,56]. The pathway from Rho to mDia1 regulates Golgi architecture and formation of Rab6-positive transport carriers, indicating that DIAPH1 participates in membrane traffic as well as cytoskeletal assembly [57]. In macrophages, the microtubule-associated protein CLIP-170 coordinates mDia1 and actin reorganization during complement receptor-mediated phagocytosis, supporting a role for DIAPH1 in trafficking dependent immune cell responses [58]. Such properties are pertinent to RAGE, because a receptor that responds to persistent tissue-damage signals is likely to rely on a partner that integrates actin remodeling, microtubule behavior, and membrane traffic into a single cellular response [57,58]. Loss of mDia1 impairs neutrophil actin polymerization, polarization, and chemotactic migration and disrupts activation of the LARG, RhoA, and ROCK signaling axis during chemotaxis [59]. In dendritic cells, the pathway from Rho to mDia1 is required for adhesion, migration, and T cell stimulation, indicating that DIAPH1 shapes both immune cell motility and immune synapse function [60]. In T lymphocytes, mDia1 regulates GSK3β-dependent microtubule dynamics required for migratory polarization [61]. More recent work confirmed that mDia1 supports T cell migration through complex environments, with functions distinct from those of FMNL1 [62]. In neutrophils, loss of mDia1 impairs CD11b endocytosis and increases endothelial adhesion, altering vascular interactions in vivo [63]. These observations show why DIAPH1 serves as an intracellular amplifier of inflammatory signaling, with functions other than only those of an accessory cytoskeletal protein [59,60,61,62,63]. Chemical biology studies showed that the cytoplasmic tail of RAGE requires interaction with DIAPH1 for ligand-stimulated signal transduction and that this interaction can be disrupted by small molecules that competitively inhibit it [64]. Biophysical studies showed that DIAPH1 influences nanoscale clustering and lateral diffusion of RAGE, suggesting that DIAPH1 contributes not only to downstream signaling but also to receptor organization at the membrane [65]. More recent work showed that a selective small molecule antagonist of RAGE–DIAPH1 suppressed DIAPH1 activation and inhibited inflammatory responses in human macrophages [66].

In vascular smooth muscle cells and in vivo models, mDia1 facilitates vascular remodeling and links oxidative and signaling pathways, placing this formin within processes central to chronic cardiometabolic injury [67]. In the heart, DIAPH1 expression increases after experimental ischemia and reperfusion, and genetic deletion of *Diaph1* causes smaller infarcts and improved contractile function [68]. In that setting, silencing *Diaph1* reduces actin polymerization and lowers serum response factor-regulated gene expression while favorably modifying calcium transporter expression in cardiomyocytes [68]. More recently, DIAPH1 was shown to interact with Mitofusin-2 in cardiomyocytes, endothelial cells, and macrophages. Through this interaction, it shortens the distance between mitochondria and the sarcoplasmic reticulum or endoplasmic reticulum and regulates mitochondrial turnover, mitophagy, and oxidative stress during ischemic or hypoxic stress [69].

### 2.4. Cellular Effects of AGE–RAGE Signaling and Signal Propagation Mediated by DIAPH1

In this section, we organize cellular effects by level of mechanistic support. Some are directly linked to AGE–RAGE signaling, some include evidence for DIAPH1 participation; others are mechanistic hypotheses.

Activation of RAGE by AGEs induces oxidative injury via NADPH oxidase. Pharmacological inhibition of this enzyme reduces damage observed in vivo [70]. AGEs reduce nitric oxide (NO) bioavailability in vitro and in vivo and impair vasodilatation mediated by the endothelium in experimental diabetes [71]. A related loss of endothelial homeostasis has been described in human coronary artery endothelial cells, where AGEs suppress endothelial nitric oxide synthase expression through oxidative stress, thus connecting carbonyl stress with impaired signaling mediated by NO in the coronary circulation [72].

The effects of AGEs on the endothelium are clearly proinflammatory and prothrombotic. Albumin modified by AGEs induces tissue factor in a RAGE-dependent manner. This effect is partly controlled by NF-κB, suggesting NF-κB helps drive the prothrombotic program triggered by AGE–RAGE binding [73]. Exposure of endothelial cells to AGEs likewise up-regulates MCP-1, ICAM-1 and VCAM-1, and increases monocyte adhesion and ROS [74]. Repair processes are also compromised. In endothelial progenitor cells, AGE exposure activates RAGE together with NADPH oxidase and c-Jun N-terminal kinase signaling, increases apoptosis, and suppresses proliferation [75]. AGEs also increase vascular endothelial growth factor (VEGF) expression and vascular hyperpermeability through mechanisms mediated by ROS, and this effect is inhibited by pigment epithelium factor [76].

Barrier failure is a recurrent consequence of AGE signaling in the vascular system. In endothelial monolayers, AGEs increase permeability through the RAGE and Rho pathway, promote intercellular gap formation, and induce actin reorganization associated with cell contraction [77]. This disturbance is exacerbated by β-catenin phosphorylation, which leads to upregulation of ADAM10 [78]. Consistent with this pattern, AGEs activate RhoA and ROCK, which increases moesin phosphorylation and stress fiber formation, leading to higher endothelial permeability [79]. VE-cadherin internalization also occurs through phosphorylation of moesin at Thr558 [80]. In human endothelial cells, mDia1 appears to be a crucial intracellular mediator of these changes. Downregulation of mDia1 attenuates AGE-induced endothelial hyperpermeability, whereas overexpression intensifies this response [81]. Comparable alterations have been reported in brain microvascular endothelial cells, where AGEs increase permeability through VEGF expression induced by ROS and are accompanied by prolonged oxidative stress and impaired mitochondrial respiration, both of which coincide with barrier disruption [82,83]. Within the peripheral nervous system, the same pathogenic logic extends to the blood–nerve barrier. AGEs induce basement membrane hypertrophy and barrier dysfunction, and these effects depend on increased autocrine signaling of VEGF and transforming growth factor beta (TGF-β) in pericytes, which links endothelial and pericyte injury to nerve ischemia and increased neural susceptibility [84].

AGE signaling also contributes to fibrotic remodeling in several tissues. AGEs activate fibrotic signaling directly without needing TGF-β receptor signaling or secondary cytokines. Renal tubular cells respond to AGEs via Smad3 to produce connective tissue growth factor and collagen [85]. Matrix accumulation mediated by connective tissue growth factor is likewise induced in renal fibrosis predominantly through a pathway independent of TGF-β [86]. In the heart, AGEs stimulate cardiac fibroblast growth and collagen synthesis by modulating KCa3.1 channels. Blocking RAGE or inhibiting downstream kinases (extracellular signal-regulated kinases, p38, phosphatidylinositol 3-kinase (PI3K), and Akt) reduces these effects [87]. Evidence from heart failure models indicates that the AGE–RAGE axis promotes myocardial fibrosis by activating cardiac fibroblasts through autophagy, thereby linking metabolic stress to structural remodeling of the myocardium [88]. Fibroblasts are not the only cells affected by this profibrotic mechanism. Genetic deletion of RAGE reduces cardiac fibrosis by repressing endothelial-to-mesenchymal transition mediated by excessive autophagy, which extends the involvement of this pathway to the endothelial compartment as well [89].

**Figure 3:**
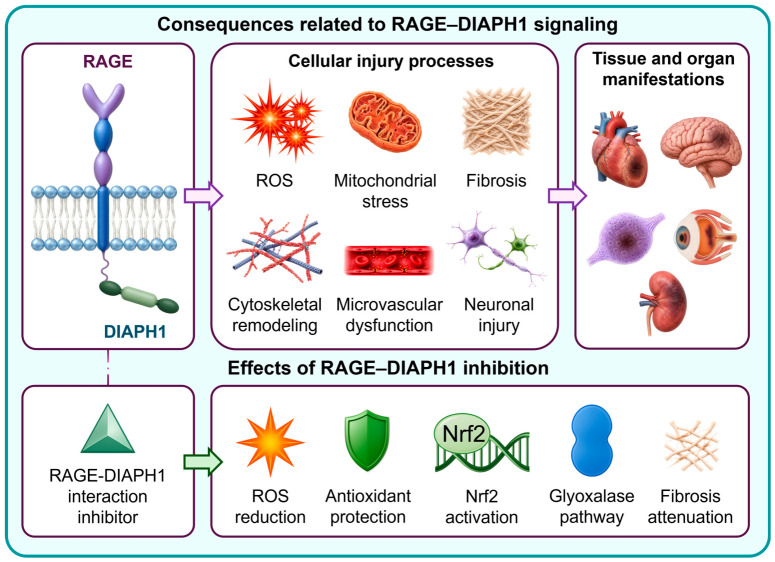
Selected cellular injury processes and tissue or organ manifestations associated with RAGE signaling and potential DIAPH1 involvement in diabetic myocardial and neuronal injury. Following RAGE activation, interaction of the RAGE cytoplasmic domain with DIAPH1 can contribute to intracellular signal propagation and injury responses. The figure shows selected downstream processes, including ROS production, mitochondrial stress, fibrosis, cytoskeletal remodeling, microvascular dysfunction, and neuronal injury. These processes may contribute to tissue and organ manifestations of diabetic complications, including cardiac, cerebral, autonomic, retinal, and renal involvement. The lower panel shows selected modulatory strategies acting after RAGE activation, including inhibition of the RAGE–DIAPH1 interaction, ROS reduction, antioxidant protection, Nrf2 activation, support of the glyoxalase pathway, and attenuation of fibrotic remodeling. Direct evidence for DIAPH1 involvement differs across tissues and remains limited in human diabetic myocardial and autonomic disease; therefore, the scheme should be interpreted as a conceptual summary of selected RAGE-associated processes with potential DIAPH1 involvement, not as evidence that all depicted effects are uniformly dependent on DIAPH1.

Figure 3 summarizes the downstream cellular and tissue consequences attributed to AGE–RAGE signaling and, where supported, to signal propagation involving DIAPH1. It also indicates potential post-receptor modulation points that reduce ROS, boost antioxidant levels, activate Nrf2, support the glyoxalase pathway, and limit fibrotic remodeling.

## 3. Neurocardiac Manifestations and Biomarkers of AGE–RAGE–DIAPH1 Signaling in T2DM

### 3.1. Myocardial, Microvascular, and Autonomic Consequences in T2DM

Cardiac involvement in T2DM is recognized as a diabetic myocardial disorder. It represents a heart-disease phenotype that develops independently of ischemic heart disease, valvular disease, or chronic pressure overload [13]. Early disease often includes left ventricular diastolic dysfunction with preserved ejection fraction and subclinical systolic impairment detected by myocardial strain imaging [90]. Greater myocardial extracellular volume and lower stress myocardial blood flow are independently associated with worse diastolic function in T2DM [91]. Reduced coronary flow reserve correlates with impaired left ventricular filling pressure in T2DM [92]. Poor glycemic control is also associated with a higher prevalence of coronary microvascular dysfunction with depressed flow reserve [93].

AGEs induce autophagy in cardiomyocytes through RAGE signaling [94] and reduce the calcium transient while increasing ROS and NO generation [95]. Chronic diabetes increases AGE adduct formation on the sarcoplasmic reticulum Ca^2+^ ATPase, providing a structural basis for abnormal Ca^2+^ reuptake in the diabetic heart [96]. AGE exposure induces mitochondrial dysfunction and apoptosis in cardiomyocytes through pathways involving protein kinase C delta (PKCδ) [97] and c-Jun N-terminal kinase (JNK) [98]. Myofilament glycation also impairs contractility by restricting tropomyosin movement [99]. Autonomic dysfunction in T2DM is associated with reduced HRV and abnormal cardiovascular reflexes [100] and is independently linked to subclinical myocardial dysfunction [101] and left ventricular diastolic impairment [102]. Skin biopsy specimens from diabetic neuropathy showed stronger vascular RAGE staining and higher RAGE mRNA in more severe disease [103]. In adult sympathetic neurons, high glucose increases RAGE expression and oxidative stress markers, while AGEs, S100 proteins, and HMGB1 reproduce ROS generation and inactivation of neuronal nicotinic acetylcholine receptors. Antioxidants, antibodies against RAGE, and genetic loss of the receptor prevent these effects [104]. Superior cervical ganglion neurons from mice with diabetes induced by streptozotocin (STZ) showed swollen mitochondria with disrupted cristae, and these changes were attenuated by RAGE deficiency. In cultured neurons, high glucose induced mitochondrial fragmentation and trafficking abnormalities in a manner dependent on RAGE. RAGE was also detected in fractions enriched in mitochondria and colocalized with a mitochondrial marker, implicating this pathway in direct mitochondrial injury in autonomic neurons [20]. This is consistent with recent synthesis placing AGE–RAGE signaling among the molecular pathways implicated in diabetic neuropathy and its progression [105]. High glucose also potentiates TRPV1 currents and depolarization responses in dorsal root ganglion neurons through mechanisms that require RAGE, calcium influx, ROS, protein kinase C, and Src signaling [41]. Peripheral neural injury is also shaped by endothelial dysfunction, because increased endoneurial capillary permeability is sufficient to induce neuropathy in diabetes-related models [106].

These myocardial, microvascular, and autonomic abnormalities should not be viewed as isolated endpoints. Autonomic dysfunction may aggravate myocardial disease by altering heart rate control, chronotropic reserve, coronary vasomotor regulation, and myocardial oxygen demand. Coronary microvascular dysfunction and myocardial fibrosis may, in turn, further disrupt autonomic cardiovascular regulation by reducing perfusion reserve and altering ventricular loading conditions. In T2DM, CAN has been associated with subclinical myocardial dysfunction and left ventricular diastolic impairment, and coronary microvascular dysfunction together with diffuse myocardial fibrosis has been associated with impaired diastolic performance [91,92,93,101,102]. Within this integrated phenotype, AGE–RAGE signaling provides a plausible shared mechanism linking endothelial barrier dysfunction and fibrotic remodeling. Direct evidence that DIAPH1 drives human diabetic myocardial disorder or cardiovascular autonomic neuropathy remains limited. Small-molecule antagonism of the interaction between the RAGE cytoplasmic domain and DIAPH1 reduced diabetic complications in mice [12], and a newer antagonist inhibited DIAPH1 activation mediated by RAGE and inflammatory signaling in human macrophages [66].

### 3.2. Biomarkers Related to AGE–RAGE–DIAPH1 Signaling in T2DM

Biomarkers and readouts relevant to this review can be grouped into three categories. The first includes axis-proximal markers, such as circulating AGEs, specific AGE adducts, soluble RAGE isoforms, AGEs/cRAGE ratio, and skin AGE measurements. The second includes downstream consequence markers, such as IL-6, CRP, N-terminal pro-B-type natriuretic peptide (NT-proBNP), and high-sensitivity cardiac troponin T (hs-cTnT), which reflect inflammation, myocardial stress, or tissue injury but are not specific to AGE–RAGE–DIAPH1 signaling. The third includes tissue and functional readouts, such as myocardial extracellular volume, native T1 mapping, heart rate variability, cardiovascular reflex tests, coronary flow reserve, diastolic function, and strain imaging. This classification is important because pathway proximity does not equal diagnostic specificity, and none of the currently available clinical readouts directly measure DIAPH1 activity.

Circulating AGEs and soluble RAGE isoforms are the most promising biomarker candidates. Among individuals with T2DM, higher levels of these markers, particularly a higher AGEs to cRAGE ratio, were associated with all-cause mortality and cardiovascular complications during long-term follow-up [107]. At the time of diagnosis, combined assessment of markers of advanced glycation, receptor expression and soluble receptor levels, oxidative and endothelial injury markers, and noninvasive vascular measures linked the AGE–RAGE axis to early cardiovascular risk [108]. In T2DM, IL-6 and its change over time were associated with cardiovascular and kidney outcomes, and higher IL-6 was associated with incident heart failure [109,110]. Baseline and serial CRP measurements improved prediction of cardiovascular events and mortality in high-risk T2DM [111], while in early T2DM high-sensitivity CRP was more strongly related to all-cause mortality than to first cardiovascular events [112]. Oxidative stress biomarkers are difficult to position clinically, because in diabetic patients, who were followed longitudinally, they predicted progression of peripheral and cardiac autonomic nerve dysfunction [113], but in a separate cohort circulating redox biomarkers did not improve prediction of adverse cardiovascular events [114]. In T2DM, NT-proBNP predicted death and cardiovascular events and, among NT-proBNP, hs-cTnT, IL-6, and high-sensitivity CRP, it provided the strongest incremental value for heart failure prediction beyond clinical variables and the other biomarkers [115,116]. High-sensitivity cardiac troponin T had prognostic value in T2DM also without clinically apparent cardiovascular disease [117]. In another analysis, NT-proBNP and hs-cTnT were each associated with adverse cardiovascular events, and the combination identified a subgroup at particularly high risk [118].

In asymptomatic patients with T2DM and preserved myocardial systolic strain, cardiovascular magnetic resonance detected increased native T1 and extracellular volume, consistent with early extracellular expansion and diffuse fibrosis [119]. Myocardial fibrosis was associated with functional and metabolomic parameters, which supports its biological relevance as a tissue phenotype in diabetes [120]. Cardiac autonomic phenotyping also requires functional assessment. HRV remains one of the most informative measures because it directly captures autonomic imbalance, and both prediabetes and T2DM have been associated with lower HRV [121]. However, HRV should not be interpreted as a stand-alone, uniformly standardized biomarker, because its values depend on recording conditions, respiratory influences, age, medication use, glycemic state, comorbid cardiovascular disease, and the specific analytical indices used. Accordingly, HRV is most informative when interpreted together with cardiovascular autonomic reflex testing and the broader clinical context [16,17,18]. Noninvasive skin AGE measurements were associated with distal sensorimotor polyneuropathy and CAN, including both sympathetic and parasympathetic involvement [122]. Skin AGE levels were also higher in the presence of CAN and correlated with the degree of autonomic impairment [123], and a later study reported a similar association [124]. Skin autofluorescence extended these observations to cardiovascular outcomes, predicting new cardiovascular disease and mortality in one prospective cohort [125] and being associated with a higher risk of cardiovascular events in another [126].

Representative biomarkers and functional readouts related to the AGE–RAGE–DIAPH1 axis in T2DM are summarized in Table 2.

## 4. Pharmacological Modulation of the AGE–RAGE–DIAPH1 Axis

Therapeutic targeting of the AGE–RAGE–DIAPH1 axis has been considered at several levels. These include suppression of AGE formation, enhancement of dicarbonyl detoxification, neutralization of accumulated ligands, blockade of RAGE, and inhibition of intracellular RAGE–DIAPH1 signaling. RAGE lacks kinase activity and depends on cytoplasmic binding partners for signaling. Foundational studies showed that the cytoplasmic domain of RAGE binds DIAPH1 and that this interaction is required for ligand induced migration and activation of Rac1, Cdc42, and AKT, establishing intracellular RAGE coupling as a pharmacological target [10,127]. Most pharmacological evidence in this area comes from diabetic nephropathy, vascular injury, wound healing, retinopathy, and inflammatory models. Therefore, these studies should be interpreted primarily as evidence that different levels of the AGE–RAGE pathway are pharmacologically modifiable, not as direct proof of efficacy in diabetic myocardial disorder or cardiovascular autonomic neuropathy. Neurocardiac relevance is strongest when studies examine myocardial structure or function, autonomic or sensory neuropathy, coronary or endothelial dysfunction, mitochondrial injury, or cardiac fibrosis.

In a large randomized clinical trial in type 1 diabetic nephropathy, pimagedine, an inhibitor of AGE formation, did not significantly improve the primary renal endpoint. However, it slowed the decline in glomerular filtration rate, reduced proteinuria, and was associated with less retinopathy progression, with safety concerns [128]. Pyridoxamine reduced the rise in serum creatinine in combined phase 2 studies of overt diabetic nephropathy, with the clearest effect in participants with less advanced renal impairment [129]. In a trial of the pyridoxamine derivative Pyridorin in proteinuric T2DM diabetic nephropathy, one year of treatment did not delay the progression of renal function loss [130]. In another trial in abdominal obesity, pyridoxamine lowered MGO and markers of glycation and endothelial activation, while not improving insulin sensitivity or vascular function [131]. A similar pattern was seen with benfotiamine. Although it reduced endothelial dysfunction and oxidative stress after a meal rich in AGEs in individuals with T2DM [132], 12 months of treatment did not improve measures of neuropathy in a T2DM trial with symptomatic polyneuropathy [133]. An AGE aptamer blocked the progression of experimental diabetic nephropathy [134] and, in diabetic retinopathy, prevented electroretinographic abnormalities without altering glycemia [135]. A RAGE aptamer attenuated the development and progression of experimental diabetic nephropathy, and in obese diabetic mice RAGE aptamer treatment suppressed renal tubular damage and improved insulin resistance [136,137]. A decoy strategy using soluble RAGE suppressed accelerated diabetic atherosclerosis in apoE-deficient mice, while RAGE deficiency attenuated accelerated atherosclerosis in diabetic mice [138,139]. Additional support for receptor-level intervention came from a study showing that low-molecular-weight heparin binds RAGE and attenuates albuminuria and structural renal injury in diabetic mice, linking direct receptor antagonism to organ protection in diabetic nephropathy [140]. In diabetes induced by STZ, a RAGE fusion protein prevented early diabetic retinopathy and reduced tactile allodynia, indicating that receptor decoy approaches may affect both vascular and neural phenotypes [141]. Active immunization against RAGE also attenuated the progression of diabetic kidney disease in mice, extending extracellular RAGE targeting from passive blockade toward immunological strategies [142].

Experimental RAGE blockade exerted renoprotective effects in diabetic nephropathy and was associated with induction of the renal angiotensin II type 2 receptor [143]. Blockade of RAGE restored effective wound healing in diabetic mice, and systemic treatment with a RAGE blocking antibody improved wound healing in diabetic pigs [144,145]. Azeliragon ameliorated diabetic neuropathy in mice with diabetes induced with STZ, without lowering blood glucose, and in leptin deficient obese mice the selective RAGE inhibitor TTP488 improved diabetic bladder dysfunction and reduced molecular indices linked to AGE–RAGE signaling [146,147]. In diabetic mice, liraglutide reduced renal RAGE expression, limited expansion of bone marrow myeloid progenitors, promoted macrophage polarization toward a reparative phenotype, and attenuated kidney injury. Thus, some approved agents may dampen RAGE-linked injury as part of a protective program and not a direct receptor antagonism alone [148]. Small molecule antagonism of the interaction between the RAGE cytoplasmic domain and DIAPH1 reduced diabetic complications in mice, including impaired wound healing, ischemic damage, and kidney injury [12]. Pharmacological antagonism of RAGE signaling also reduced adiposity and improved metabolic profile while enhancing thermogenic and mitochondrial function in adipose tissue in mice [149]. A selective small molecule antagonist of the RAGE–DIAPH1 interaction suppressed DIAPH1 activation, reduced inflammatory responses in human macrophages, suppressed delayed type hypersensitivity, and accelerated diabetic wound healing [66]. Recent work has shown that a brain-targeted blocking peptide directed against ctRAGE–RIPK1 attenuated cognitive deficits associated with diabetes in mice, reduced neuroinflammation, and improved neuronal integrity. Although this study did not target DIAPH1 directly, it supports the concept that pathogenic RAGE signaling can be interrupted at the level of defined intracellular protein interactions [150]. In db/db mice, THBru improved systolic and diastolic function and reduced cardiac remodeling through inhibition of RAGE-dependent inflammation [151]. Recent mechanistic work has extended this axis to autonomic neurons. In cultured autonomic neurons exposed to hyperglycemia, RAGE mediated mitochondrial fragmentation, trafficking abnormalities, and oxidative injury, while genetic deletion of RAGE attenuated these changes [20]. These observations support the view that the AGE–RAGE–DIAPH1 axis is not restricted to renal and vascular injury but may also participate directly in the neural and myocardial pathology that underlies diabetic neurocardiac complications. Many pharmacological studies of AGE–RAGE or RAGE–DIAPH1 modulation have been conducted in renal, vascular, retinal, and wound-healing models. In this review, these studies are interpreted primarily as mechanistic evidence for axis modulation rather than as definitive evidence of established efficacy in diabetic myocardial disorder or cardiovascular autonomic neuropathy.

Clinical translation has remained uneven. Preclinical studies have been more consistent, particularly for approaches that limit RAGE engagement or block intracellular RAGE signaling. Table 3 summarizes selected pharmacological strategies acting at different levels of this pathway, including AGE formation, RAGE signaling, and intracellular protein interactions involved in RAGE signal propagation.

## 5. Natural Compounds and Nutraceuticals Targeting AGE–RAGE and Associated Pathways

Natural compounds and nutraceutical preparations are best interpreted as indirect modulators of AGE formation, RAGE biology, and downstream oxidative or inflammatory injury and not as DIAPH1-directed interventions. Their reported actions include reactive carbonyl trapping, suppression of AGE formation, reduction in RAGE expression, modulation of soluble RAGE release, attenuation of NF-κB-mediated inflammatory signaling, activation of antioxidant pathways, and reduction in fibrotic remodeling [74,152,153]. The clearest evidence at present comes from in vitro studies that examine carbonyl trapping and inhibition of AGE formation. In vitro, pomegranate phenolics inhibited AGE formation through scavenging of reactive carbonyl species [152]. In adults with T2DM, pomegranate juice reduced lipid peroxidation but did not lower circulating AGEs [154]. Chlorogenic acid inhibited AGE formation and AGE-protein crosslinking in vitro [155]. Aged garlic extract inhibited AGE formation in vitro more effectively than fresh garlic extract and reduced oxidative damage associated with glycation [156]. Thymoquinone from black cumin inhibited early glycation products, products formed after the Amadori stage, products formed through pathways mediated by MGO, and late-stage AGE formation in several in vitro systems [157]. Rooibos is another relevant example because it shows methylglyoxal trapping and antiglycation activity in vitro. Aspalathin, orientin, and isoorientin have been identified as major contributors to methylglyoxal trapping and inhibition of AGE formation [158,159]. Green tea extracts rich in EGCG increased soluble RAGE secretion and interfered with S100A12–RAGE signaling, providing a receptor-level mechanism for the antiglycation chemistry described above [160]. These examples differ from simple antiglycation chemistry because they act more closely with ligand availability, receptor expression, or RAGE signaling. A further mechanism within this group involves the reduction in tissue RAGE expression under diabetic conditions. Resveratrol reduced hepatic RAGE expression, as well as total oxidant status and malondialdehyde levels, in diabetic rats [153]. A smaller group of studies has transferred these observations to animal models and early clinical settings. In a randomized, placebo-controlled trial in patients with diabetic nephropathy, resveratrol reduced albuminuria, although short-term treatment did not clearly change estimated glomerular filtration rate or serum creatinine [161]. In overweight and obese subjects, combined trans-resveratrol and hesperetin increased glyoxalase 1 and was associated with improvement in insulin resistance, dysglycemia, blood pressure, dyslipidemia, and low-grade inflammation [162]. In diabetic rats, hesperetin inhibited the AGE–RAGE axis, reduced renal levels of inflammatory mediators, and improved renal structure and function by activating the Nrf2 antioxidant response element and the glyoxalase 1 pathway [163]. Renal AGE formation, CML accumulation, and AGE receptor expression were reduced by Korean red ginseng in diabetic rats [164], and American ginseng subjected to heat processing suppressed diabetic renal injury, associated with reduced AGE formation and lower RAGE activation [165]. A smaller group of compounds has been studied in organ injury phenotypes that are more relevant to the present review. In diabetic rats, quercetin ameliorated kidney injury and suppressed HMGB1, RAGE, and NF-κB signaling [166]. In Zucker diabetic fatty rats, quercetin alleviated diastolic dysfunction, reduced myocardial collagen content, and suppressed signaling that promotes hypertrophy [167]. Curcumin alleviated diabetic retinal injury, reduced oxidative stress, and transcriptomic analysis linked these effects to inhibition of AGE–RAGE signaling in diabetic retina [168]. The ginger compound zerumbone attenuated retinal inflammation and angiogenesis in diabetic rats by blocking the AGEs–RAGE–NF-κB pathway, and 6-shogaol suppressed oxidative and inflammatory responses induced by AGEs in human cells [169,170]. In diabetic rats, hawthorn leaf flavonoids attenuated myocardial injury while lowering oxidative stress, TNF-α, PKC-α, and NF-κB signaling [171]. In mesangial cells and in experimental diabetic nephropathy, berberine exerted renoprotective effects by regulating the AGE–RAGE pathway, with improvements in biochemical and histopathological indices of renal injury [172]. In hyperglycemic mice, berberine suppressed AGE formation and attenuated diabetic retinopathy [173]. Cinnamaldehyde ameliorated diabetic nephropathy in a rat model and reduced renal accumulation of AGEs, RAGE, and CML together with inflammatory mediators linked to the AGE–RAGE axis [174]. Among these compounds, sulforaphane stands out because its effects have been examined at multiple levels of experimental design [175]. It inhibited inflammation in endothelial cells exposed to AGEs and in the rat aorta, partly through suppression of RAGE expression and oxidative stress, with parallel reductions in monocyte chemoattractant protein 1, intercellular adhesion molecule 1, and vascular cell adhesion molecule 1 [74]. Sulforaphane inhibited cardiac fibrosis induced by the AGE–RAGE axis through reduction in oxidative stress [176]. In db/db mice, oral sulforaphane attenuated a myocardial injury phenotype and improved abnormalities of myocardial lipid metabolism. This extends its effects from vascular and antifibrotic settings to a wider cardiometabolic phenotype relevant to diabetic myocardial injury [177]. Several fungal compounds reduce oxidative and inflammatory injury in diabetes, but only a few are linked directly to AGE formation or RAGE signaling. *Lactarius deterrimus* extract reduced systemic glycation markers and pancreatic islet immunoreactivity for CML and RAGE in rats with diabetes induced with STZ [178]. Extracts from *Lactarius deterrimus* and *Castanea sativa* reduced protein glycation, activation of RAGE by CML, NF-κB activity, and hepatorenal injury in diabetic rats [179]. Other fungal compounds, including *Ganoderma lucidum* and cordycepin derived from *Cordyceps militaris*, show antioxidant, anti-inflammatory, antifibrotic, or ferroptosis-modulating effects in diabetic kidney models. Still, their direct connection with AGE formation, RAGE signaling, or DIAPH1 remains weaker [180,181,182]. Comparison across studies is difficult because preparations, extraction procedures, doses, and study designs are highly heterogeneous. Purified compounds may have limited oral bioavailability, and doses used in rodents are not easily transferred to humans. Most clinical trials are too short to determine if changes in oxidative or inflammatory biomarkers translate into lower AGE levels or sustained organ protection [154,161,176]. No natural compound discussed in this section has been shown to inhibit DIAPH1 or the RAGE–DIAPH1 interaction directly. Selected natural compounds and nutraceutical approaches with reported effects on AGE formation, RAGE biology, and diabetic tissue injury are summarized in Table 4.

## 6. Translational Gaps and Future Directions

The principal translational gap concerns the difference between a biologically coherent signaling concept and a clinically validated disease mechanism. AGE formation, RAGE ligand accumulation, and RAGE-dependent oxidative and inflammatory signaling are well supported across vascular and tissue injury related to diabetes. DIAPH1, however, has not yet been sufficiently validated in human diabetic myocardial or neuronal disease. Key unanswered questions include if DIAPH1 biology and interaction with RAGE changes in human diabetic heart and autonomic system. A second limitation is cell-type specificity. The AGE–RAGE axis may operate differently across various cell types. Future studies should therefore avoid treating diabetic myocardial and neuronal injury as homogeneous endpoints. Human tissue studies, cell cultures, single-cell approaches, spatial transcriptomics, and proteomics could help determine if DIAPH1 biology or interaction with RAGE is altered in disease-relevant cell populations. Such validation would be especially valuable in diabetic myocardium, coronary microvasculature, and autonomic system, where cell-type-specific AGE–RAGE–DIAPH1 activity cannot be inferred reliably from tissue measurements alone. A third gap concerns biomarkers and therapeutic translation. Current readouts may support phenotyping, but none quantify DIAPH1 activity. Similarly, RAGE–DIAPH1 antagonists provide a rational strategy for blocking intracellular signal propagation downstream of RAGE, but their current evidence base remains mainly preclinical or translational. Future studies should examine if the inhibition of the RAGE–DIAPH1 interaction modifies detrimental neurocardiac consequences.

## 7. Conclusions

The AGE–RAGE–DIAPH1 axis provides a coherent mechanistic concept for understanding how carbonyl stress and inflammatory signaling may be involved in diabetic myocardial and neuronal injury. The RAGE cytoplasmic domain interacts with DIAPH1, a protein that supports intracellular signaling. This interaction contributes to an increase in oxidative stress and immune response, and subsequently cytoskeletal remodeling and fibrosis.

However, a credible mechanistic concept does not directly translate into a validated clinical target. The AGE–RAGE component of the pathway is supported more strongly than DIAPH1-specific involvement in human diabetic myocardial or neuronal disease. DIAPH1 should be viewed as a promising intracellular amplifier and translational research hypothesis rather than as an established biomarker or therapeutic target. Future studies should determine if DIAPH1 is altered in disease-relevant human tissues and if RAGE–DIAPH1 inhibition modifies clinically meaningful myocardial, vascular, or neuronal outcomes in T2DM.

## Figures and Tables

**Table 1:** Hierarchy of evidence supporting components of the AGE–RAGE–DIAPH1 concept in diabetic myocardial and neuronal injury.

Domain	Evidence Rating	Main Interpretation
AGE formation and carbonyl stress in diabetes	★★★★★	Strong biochemical, experimental, and clinical support
AGE–RAGE signaling in endothelial dysfunction and barrier failure	★★★★	Strong experimental support with clear relevance to vascular complications
AGE–RAGE signaling in myocardial and neuronal injury	★★★	Supported by experimental cardiac, sensory neuron, and autonomic neuron studies; causal human validation remains incomplete
RAGE signaling in autonomic neuronal vulnerability and CAN-relevant mechanisms	★★★	Emerging evidence, including autonomic neuron models; direct clinical validation in CAN remains incomplete
DIAPH1 as an intracellular effector of RAGE signaling	★★★★	Strong mechanistic evidence for RAGE–DIAPH1 coupling, receptor organization, and inflammatory signaling
DIAPH1 in diabetic myocardial and neuronal injury	★	Direct human tissue evidence remains limited or absent; currently a mechanistic hypothesis requiring validation
RAGE–DIAPH1 antagonism as a therapeutic strategy	★★★	Promising preclinical and translational evidence; no clinical validation in diabetic myocardial or neuronal complications
Biomarkers and translational readouts of AGE–RAGE–DIAPH1 signaling	★★★	AGE adducts, soluble RAGE isoforms, and skin AGE measures are informative, but no clinically validated DIAPH1-specific biomarker is available
Natural compounds and nutraceutical approaches	★★	Mostly in vitro and animal evidence for AGE formation or RAGE-related pathways; no convincing direct evidence for DIAPH1 targeting

Note: Evidence ratings are qualitative author assessments of the relative strength of the available evidence and do not represent a formal grading of clinical efficacy. A higher number of stars indicates stronger and more direct support.

**Table 2:** Biomarkers and translational readouts linked to AGE–RAGE–DIAPH1 signaling.

Biomarker/ Readout	Biological Compartment	Association with Phenotype	Translational Relevance
Circulating AGE/RAGE markers			
Circulating AGEs [107]	Plasma	Mortality, cardiovascular complications	Systemic glycation burden
Circulating methylglyoxal [25,26]	Plasma	Cardiovascular events and mortality	Dicarbonyl stress marker
CML-modified proteins [27,36]	Plasma, extracellular matrix proteins	RAGE activation and inflammatory signaling	Mechanistic AGE marker
Soluble RAGE isoforms [107]	Plasma	Cardiovascular outcomes	Soluble RAGE compartment
AGEs/cRAGE ratio [107]	Plasma	Mortality and complications	Integrated ligand–receptor balance
Skin AGE readouts			
Skin AGEs [122,124]	Skin	Neuropathy and CAN	Non-invasive screening
Skin autofluorescence [125,126]	Skin	Cardiovascular events and mortality	Long-term AGE burden
Tissue RAGE and DIAPH1-related readouts			
RAGE expression in diabetic neuropathy [103]	Skin biopsy specimens	Severity of diabetic neuropathy	Local RAGE activation
RAGE in sympathetic/autonomic neurons [20,104]	Adult sympathetic neurons; superior cervical ganglion neurons	Oxidative stress, mitochondrial abnormalities	Neural mechanistic readout
RAGE–DIAPH1 interaction/DIAPH1 activation [10,12,66]	Experimental cells; human macrophages; diabetic mouse models	Intracellular RAGE signal propagation	Most axis-specific readout

Note: The table includes markers and translational readouts that directly reflect AGE burden, dicarbonyl stress, the soluble or tissue RAGE compartment, or intracellular RAGE–DIAPH1 signaling. Broader inflammatory, cardiac injury, imaging, microvascular, and autonomic functional markers were not included because they are not specific to the AGE–RAGE–DIAPH1 axis.

**Table 3:** Selected pharmacological strategies targeting AGE formation, RAGE signaling, or intracellular protein interactions involved in RAGE signal propagation in T2DM and related complications.

Agent/Strategy	Proposed Mechanism	Main Reported Effects	Evidence Context
Inhibitors of AGE formation			
Pimagedine [128]	Inhibits AGE formation by limiting the generation of advanced glycation end-products.	Slower decline in GFR, reduced proteinuria, safety concerns.	Type 1 diabetic nephropathy (RCT)
Pyridoxamine [129,130]	Limits AGE formation and dicarbonyl stress by scavenging reactive carbonyl species.	Reduced creatinine rise in early disease, no effect in advanced disease.	Diabetic nephropathy (clinical trials)
Benfotiamine [132,133]	Modulates AGE formation and oxidative pathways by enhancing transketolase activity and reducing glycation intermediates.	Improved endothelial dysfunction in the short term; no long-term neuropathy benefit.	T2DM (clinical)
Ligand neutralization and decoy approaches			
AGE aptamer [134,135]	Neutralizes AGE ligands and prevents their interaction with RAGE and related receptors	Reduced nephropathy progression, retinal dysfunction	Animal models (preclinical)
RAGE aptamer [136,137]	Blocks ligand binding at the RAGE receptor	Reduced renal injury, improved insulin resistance	Diabetic mice (preclinical)
Soluble RAGE [138,139]	Acts as a soluble decoy receptor that competes with membrane RAGE for ligand binding	Reduced vascular injury and atherosclerosis	Diabetic atherosclerosis models (preclinical)
RAGE vaccination [142]	Induces antibodies against RAGE, thereby reducing receptor-mediated signaling	Attenuated diabetic kidney disease	Animal model (preclinical)
Direct RAGE antagonism			
Azeliragon/TTP488 [146,147]	Antagonizes the RAGE receptor through small-molecule inhibition	Improved neural and bladder function without glycemic effect	Diabetic neuropathy, bladder dysfunction models (preclinical)
Low molecular weight heparin [140]	Binds RAGE and interferes with receptor-mediated signaling	Reduced albuminuria and renal injury	Diabetic mice (preclinical)
Intracellular signaling inhibition			
RAGE–DIAPH1 antagonists [12,66]	Disrupt intracellular RAGE signal propagation by inhibiting the RAGE–DIAPH1 interaction	Reduced inflammation, improved wound healing, reduced diabetic complications	Animal models, human macrophages (preclinical/translational)
ctRAGE–RIPK1 peptide [150]	Blocks intracellular RAGE signaling by interfering with interactions between the RAGE cytoplasmic tail and signaling partners	Reduced neuroinflammation and cognitive impairment	Diabetic mice (preclinical)
Indirect modulators of RAGE signaling			
Liraglutide [148]	Indirectly modulates RAGE signaling by reducing RAGE expression and inflammatory activation	Reduced renal injury and inflammation	Diabetic kidney disease models (preclinical)
THBru [151]	Suppresses RAGE-associated inflammatory signaling in diabetic cardiac injury	Improved cardiac structure and function	Diabetic myocardial disorder (preclinical)

**Table 4:** Selected natural compounds and nutraceutical approaches with reported links to AGE formation, RAGE biology, and downstream diabetic tissue injury.

Agent	Proposed Mechanism	Main Reported Effects	Evidence Context
Carbonyl scavengers and AGE formation inhibitors			
Pomegranate phenolics [152,154]	Limit dicarbonyl stress and AGE formation by scavenging reactive carbonyl species	Reduced AGE formation and lipid peroxidation	In vitro assays; adults with T2DM (preclinical/clinical)
Rooibos flavonoids [158,159]	Reduce methylglyoxal availability through methylglyoxal trapping and inhibition of AGE formation	Reduced methylglyoxal reactivity and AGE formation	In vitro glycation models (preclinical)
Modulators of RAGE expression and signaling			
Green tea extracts rich in EGCG [160]	Promote soluble RAGE release and reduce S100A12–RAGE signaling	Increased soluble RAGE and reduced S100A12–RAGE signaling	Cellular model; T2DM clinical material/setting (preclinical/clinical)
Resveratrol [153,161]	Reduces tissue RAGE expression and oxidative stress under diabetic conditions	Reduced RAGE expression, oxidative stress, and albuminuria	Diabetic rats; diabetic nephropathy trial (preclinical/clinical)
Hesperetin [163]	Modulates the AGE–RAGE axis by activating Nrf2, antioxidant response pathways, and glyoxalase 1	Improved inflammatory, metabolic, and renal injury markers	Diabetic rats; overweight/obese subjects (preclinical/clinical)
Sulforaphane [74,177]	Suppresses RAGE expression, oxidative stress, and inflammatory activation; also limits cardiac fibrosis associated with AGE–RAGE signaling	Reduced inflammation, oxidative stress, fibrosis, and cardiac dysfunction	Endothelial cells, rat aorta, cardiac fibrosis and db/db mouse models; T2DM clinical biomarker studies (preclinical/clinical)
Berberine [172,173]	Regulates AGE–RAGE signaling and suppresses AGE formation under hyperglycemic conditions	Reduced glycation, inflammation, renal injury, and retinal injury	Mesangial cells and diabetic mouse models (preclinical)
Organ-protective compounds with partial AGE–RAGE linkage			
Quercetin [166,167]	Suppresses HMGB1–RAGE–NF-κB signaling and inflammatory activation	Reduced inflammation, fibrosis, and diastolic dysfunction	Diabetic rat models of renal and cardiac injury (preclinical)
Curcumin [168]	Reduces oxidative stress and inflammatory injury with transcriptomic linkage to the AGE–RAGE pathway	Reduced oxidative stress and retinal injury	Diabetic retinal injury model (preclinical)
Ginger compounds [169,170]	Suppress AGE–RAGE–NF-κB signaling and oxidative inflammatory responses induced by AGEs	Reduced retinal inflammation, angiogenesis, IL-6 expression, ICAM-1 expression, and oxidative responses	Diabetic rat retina and human cell models (preclinical)

Note: The table summarizes selected compounds with reported experimental or clinical links to AGE formation, dicarbonyl trapping, RAGE expression, soluble RAGE release, or downstream oxidative and inflammatory injury. Direct modulation of DIAPH1 or the RAGE–DIAPH1 interaction has not been demonstrated unless explicitly stated.

## Data Availability

No new data were created or analyzed in this study. Data sharing is not applicable to this article.

## Abbreviations

The following abbreviations are used in this manuscript:

AGE	advanced glycation end-product
AGEs	advanced glycation end-products
CAN	cardiovascular autonomic neuropathy
CML	Nε-carboxymethyllysine
CRP	C-reactive protein
DIAPH1	diaphanous-related formin 1
EGCG	epigallocatechin gallate
FMNL1	formin-like protein 1
GLP-1	glucagon-like peptide-1
GPX-4	glutathione peroxidase 4
GSK3β	glycogen synthase kinase 3 beta
HMGB1	high-mobility group box 1
HO-1	heme oxygenase 1
HRV	heart rate variability
hs-cTnT	high-sensitivity cardiac troponin T
IL-6	interleukin-6
MGO	methylglyoxal
NF-κB	nuclear factor kappa B
NO	nitric oxide
Nrf2	nuclear factor erythroid 2-related factor 2
NT-proBNP	N-terminal pro-B-type natriuretic peptide
RAGE	receptor for advanced glycation end-products
ROS	reactive oxygen species
STZ	streptozotocin
T2DM	type 2 diabetes mellitus
TGF-β	transforming growth factor beta
VEGF	vascular endothelial growth factor
